# Impact of Covid-19 on Household Food Waste: The Case of Italy

**DOI:** 10.3389/fnut.2020.585090

**Published:** 2020-12-02

**Authors:** Gioacchino Pappalardo, Simone Cerroni, Rodolfo M. Nayga, Wei Yang

**Affiliations:** ^1^Department of Agriculture, Food and Environment (Di3A), University of Catania, Catania, Italy; ^2^Department of Economics and Management & C3A, University of Trento, Trento, Italy; ^3^Institute of Global Food Security and Gibson Institute, Queen's University Belfast, Belfast, United Kingdom; ^4^Department of Agricultural Economics and Agribusiness, University of Arkansas, Fayetteville, AR, United States

**Keywords:** COVID-19, households, food purchasing, food waste, environmental impact

## Abstract

Covid-19 has significantly affected people's food purchasing and consumption habits. Fears of disruptions in the food supply chain have caused an increase in the quantity and type of food bought by households. However, increases in food purchases could give rise to food waste with negative ramifications for the environment in terms of greenhouse emissions and groundwater pollution. To assess whether household food waste has changed during Covid-19 lockdown, we conducted a nationwide survey of household food purchasers in Italy. Although the amount of food purchases increased during the lockdown, our results show that food waste actually decreased as people mainly bought more non-perishable food. Interestingly, concerns about the impact that the pandemic could have on the waste management system and the desire not to add pressure to the waste management system are key drivers of decreased food waste in Italy during the pandemic. Our findings seem to suggest that Italian consumers are developing a new level of awareness about food waste with potential positive impacts on the environment in terms of reducing greenhouse gas emissions and groundwater pollution.

## Introduction

Covid-19 has transformed people's daily lives amid significant shifts in people's behavior related to social distancing and stockpiling of essentials like food (1, 2). These behavioral changes may be fear-driven and due to widespread anxiety and considerable feeling of insecurity as conventional assumptions about the security of jobs, expected incomes, and the value of savings have been challenged (3, 4). The experience of negative shocks may also make people more risk-averse (5, 6).

In this framework, Covid-19 has significantly affected people's food purchasing and consumption habits. During the lockdowns in many countries around the world, restaurants and bars were shut down due to stay-at-home directives. Consequently, food sales from restaurants practically dried up during the stay-at-home orders, while food sales from grocery stores and online retailers significantly increased (7). Some food items in grocery stores have become scarce due to interruptions in food supply chains (8). The constraints imposed by the lockdown and the fear of disruptions in the food supply chain have caused a change in purchasing behavior such as an increase in food stocks at home or the quantity and type of food bought (9).

The response to the Covid-19 pandemic, from panic buying at grocery stores to restaurant closures, could affect household food waste at a time when food insecurity is on the rise. Hence, it is important to assess whether changes in food purchasing and consumption habits due to Covid-19 have had a significant impact on household food waste. Food waste is a very important environmental and economic issue given that around 88 million tons of food are wasted annually in the European Union (EU), with associated costs estimated at 143 billion euros. Food waste refers to food that goes through the food supply chain up to becoming a final product, but does not get consumed because it is discarded (10). Importantly, households contribute the most to food waste (47 million tons), accounting for 53.4% of total food waste in the EU (11).

Food waste is an important environmental issue since food production is a major contributor of greenhouse gas emissions. When food is disposed in landfills to rot, it also becomes a significant source of methane which is a potent greenhouse gas with direct consequences for global warming (12). At the same time, food waste in landfills releases toxic substances in the soil with negative consequences for groundwater (13). Since households are the most prominent contributors for food waste and given that Covid-19 has had significant impact on hunger and food insecurity, it is important to know whether households have increased or decreased food waste during the pandemic.

Past studies showed that food waste is a function of consumer demographic characteristics, and that decisions to discard food vary with contextual factors (14, 15). Hence, food waste behavior can be better understood by focusing on the practices, routines and habits of consumers given the hidden nature of the food waste issue (16). Moreover, food waste poses important ethical issues since more than 820 million people in the world have insufficient food and many more consume low-quality diets that cause micronutrient deficiencies and health problems (17). The reduction of food waste has been the subject of research, and a number of recommendations have been provided related to giving respect for food and cooking skills or by encouraging a mindset of flexibility in light of unforeseen events (18).

Early studies on Covid-19 impact on household food waste discuss that the amount of food waste may actually decrease if consumers are becoming more careful about not wasting food due to fear of food supply disruptions or difficulties in shopping (8, 19). Hence, even if panic stockpiling happens during Covid-19 pandemic encouraging many families to stockpile food (20), it is possible for household food waste to decrease if people try to use or consume everything they have bought. During emergencies or crises, people may also acquire a stronger preference for the environment (21) and therefore the experience of Covid-19 lockdown may have made people more mindful of environmental issues such as food waste. On the other hand, people may have reacted to the virus by exhibiting more individualistic behavior and less globalism leading to a resurgence of isolationism and nationalism, especially as people learn to distance themselves from social interactions (22). This will result in weaker preferences for environmental public goods with negative impacts in terms of environmental protection and reduction of greenhouse gas emissions.

The current scientific literature does not provide sufficient evidence on how households would react to emergency crises, such as pandemics, in terms of the amount of food waste produced. To meaningfully address this critical issue which has important environmental and economic consequences, we conducted a nationwide survey of household food purchasers in Italy to examine whether household food waste has changed during Covid-19 and to identify the factors that affect household food waste^1^. No other study has directly examined this issue in the EU. We conducted our survey in Italy in the month of April 2020 when strict lockdown measures were imposed by the government on the entire Italian population to counter the spread of Covid-19 infection and when all non-essential economic activities including restaurants and take-out food outlets were closed. Italy is an interesting case to study in the EU given that it was one of the countries most affected by Covid-19. In Italy, an estimated 5.5 million tons of food waste are generated each year with associated costs estimated at 15 billion euros (11). Close to 80 percent of the food waste are generated by households, with significant socio-economic and environmental effects especially in terms of global greenhouse gas emissions (23).

## Materials and Methods

### Questionnaire Development and Survey Procedures

A questionnaire was administered in Italy using the online platform Qualtrics. The sample was recruited by Dynata, a US market research company, using their Italian panel of consumers. A stratified sampling procedure was implemented which generated a representative sample of the Italian population^2^. Consumers were randomly contacted by Dynata and asked few screening questions regarding whether they are the household main food purchaser, their age, gender, and geographical area of residence. Consumers who consented to take part in the survey were allowed to participate until age, gender and geographical area of residence quotas were filled. These quotas were identified to make our sample representative of the Italian population. Respondent consent to participate in the survey was elicited through a consent form that was submitted online along with the questionnaire. Only respondents over 18 years old and who have confirmed to be the main household food purchaser were allowed to participate. Our final sample consists of 1,188 consumers. The questionnaire was administered during the lockdown in Italy, more specifically in the period from 20th−25th April 2020. The nationwide lockdown started on 9th March 2020 in Italy and ended in 14th May 2020. As part of a larger study about Italian consumers' food purchasing habits during Covid-19, respondents were asked a set of qualitative questions about whether their purchasing behavior, food expenditures, waste productions and other food-related behaviors had changed during the Covid-19 pandemic. Five points Likert-scales were used to elicit such information^3^. For example, respondents were asked whether the amount of food waste produced during the Covid-19 emergency changed and they could select among 1 (substantially decreased), 2 (mildly decreased), 3 (unchanged), 4 (mildly increased) to 5 (substantially increased), where 2. Similar questions were asked regarding the frequency of their food shopping and the amount of food purchased. Respondents were also asked to report the extent to which they agreed to a set of statements regarding possible reasons behind the reported change in food waste. Again they had the option to choose among on scale from 1 (do not agree at all) to 5 (completely agree). An example of a statement is: “My food waste production was reduced because I wanted to ease the work of people in the waste collection.” The complete questionnaire is reported in the Appendix 1.

### Behavioral Model and Estimation Procedures

To examine the factors that significantly affect household food waste in Italy during Covid-19, an ordered logit model was estimated using R. The dependent variable of our model (*WASTE*) indicated whether participants' food waste production had changed during the pandemic. We used an ordered logit model given the ordered nature of the Likert scale used to measure this variable. The ordered logit model assumes that the coefficients that describe the relationship between all pairs of groups is the same and hence we have only one coefficient associated with each variable (i.e., proportional odds assumption or the parallel regression assumption). This is one of the assumptions that differentiates the ordered logit model from a multinomial logit model. A Brant test can be used to test whether this assumption is satisfied or not. The set of independent variables included in the model is described in Table 1.^4^

**Table 1:** Summary statistics of the scores attributed by the interviewed sample units on a 5 points Likert scale.

**Dependent variable**	**Description**	**Obs**	**Mean**	**Std. dev**.	**Min**	**Max**
*WASTE*	Response on a scale from 1 (decreased substantially) to 5 (increased substantially): the amount of your food waste increased, decreased or remained unchanged?	1,188	2.237	0.999	1.000	5.000
**Independent variable**	**Description**	**Obs**	**Mean**	**Std. dev**.	**Min**	**Max**
*AM_DEC*	=1 if the amount of food purchased during the lockdown has mildly or substantially decreased, =0 otherwise	1,188	0.138	0.345	0.000	1.000
*AM_UNCHANGED* (baseline/omitted category)	=1 if the amount of food purchased during the lockdown is unchanged, =0 otherwise	1,188	0.249	0.433	0.000	1.000
*AM_INC*	=1 if the amount of food purchased during the lockdown has mildly or substantially increased, =0 otherwise	1,188	0.613	0.487	0.000	1.000
*FREQ_DEC*	=1 if the frequency of food shopping during the lockdown has mildly or substantially decreased, =0 otherwise	1,188	0.429	0.495	0.000	1.000
*FREQ_UNCHANGED* (baseline/omitted category)	=1 the frequency of food shopping during the lockdown is unchanged, =0 otherwise	1,188	0.279	0.449	0.000	1.000
*FREQ_INC*	=1 if the frequency of food shopping during the lockdown has mildly or substantially increased, =0 otherwise	1,188	0.292	0.455	0.000	1.000
*FEM*	=1 if female, =otherwise	1,188	0.540	0.499	0.000	1.000
*AGE*	Age in years	1,118	53.034	16.585	18.000	90.000
*NORTH*	=1 if lining in the north, =otherwise	1,188	0.451	0.498	0.000	1.000
*SOUTH*	=1 if lining in the south, =otherwise	1,188	0.226	0.419	0.000	1.000
*CENTER* (baseline/omitted category)	=1 if lining in the center, =otherwise	1,188	0.204	0.403	0.000	1.000
*ISLAND*	=1 if lining in islands, =otherwise	1,188	0.119	0.324	0.000	1.000
*FARM*	=1 if living in a farm, =otherwise	1,188	0.210	0.408	0.000	1.000
*HOUSEHOLD*	Number of people in the household	1,188	2.711	1.192	0.000	8.000
*LOW_INC*	=1 annual net income is < €39900, =0 otherwise	1,188	0.701	0.451	0.000	1.000
*MED_INC*	=1 annual net income is >€39900 and < €80000 =0 otherwise	1,188	0.253	0.435	0.000	1.000
*HIGH_INC*(baseline/omitted category)	=1 annual net income is >€80000 =0 otherwise	1,188	0.045	0.208	0.000	1.000

*Data were collected during the lockdown for Covid-19 in Italy (9/3/2020-14/5/2020)*.

## Results

Summary statistics of the participants are shown in Table 2. The average age of the subjects is 53.03 years. Most of the subjects in the sample are female (54%). The average household size is 2.7 and the yearly average household income of the sample is < €39,900 Euros.

**Table 2:** Socio-demographic characteristics of the interviewed samples (1,188 obs).

**Variable**	**Mean**
Gender (%)	
Female	54.0
Male	46.0
Age	
Years	53.03
Household (members)	
Members	2.76
Income (%)	
Low income	70.7
Med income	25.3
Hig income	4.0
Which part of Italy do you live in? (%)	
- South	22.6
- North	45.1
- Center	20.4
- Islands	11.9

*Data were collected during the lockdown for Covid-19 in Italy (9/3/2020-14/5/2020)*.

About 33 and 16% of the sample reported that the amount of produced food waste decreased substantially and decreased mildly, respectively, during the lockdown. About 45% declared that it was unchanged, while only 5 and 1% indicated that their food waste increased mildly and substantially, respectively.

**Figure 1:**
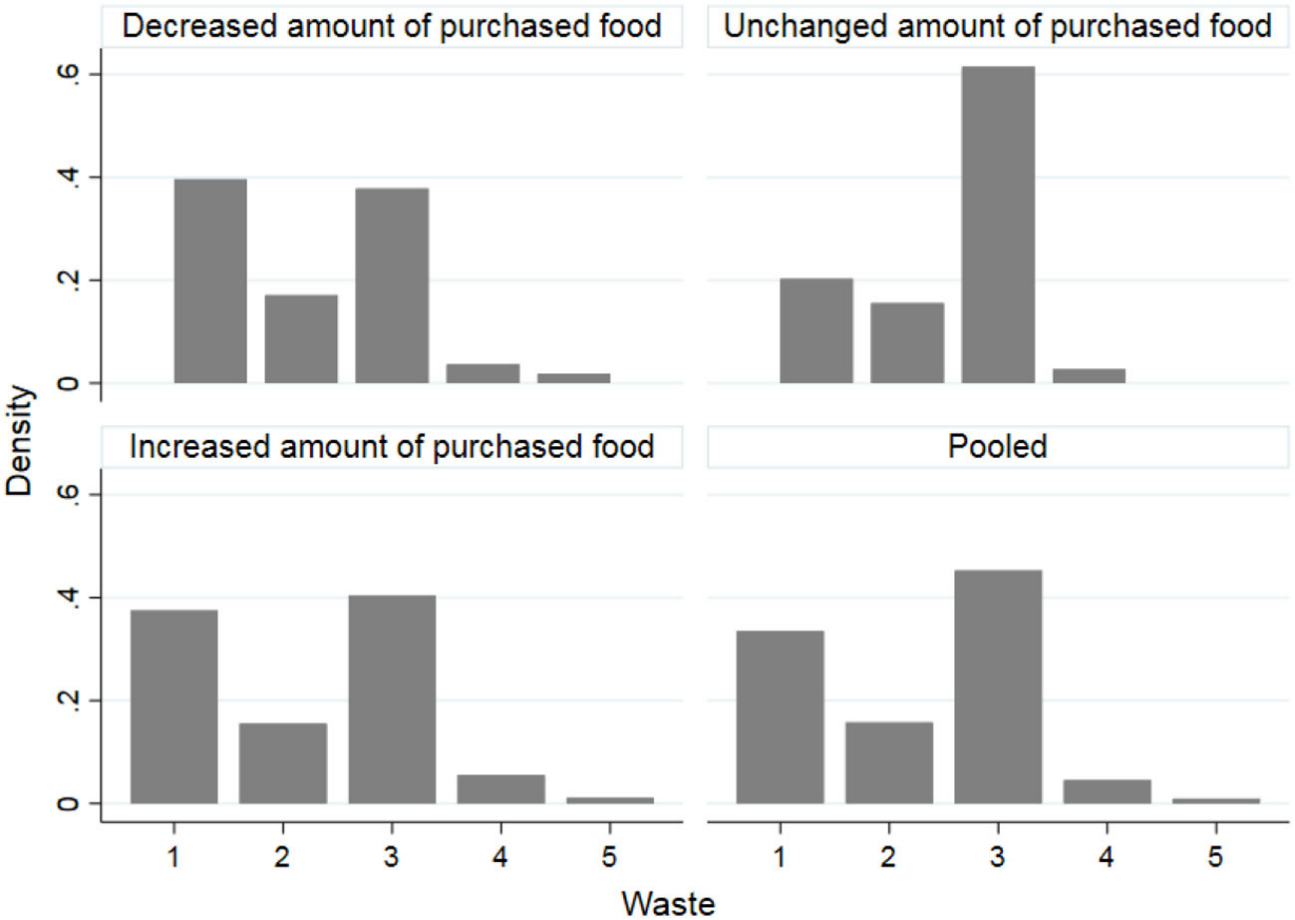
Relationships between food waste changes and amount of purchased food during the lockdown for COVID-19 in Italy. 1 = food waste substantially decreased, 2 = food waste mildly decreased, 3 = food waste unchanged, 4 = food waste mildly increased, 5 = food waste substantially decreased.

**Figure 2:**
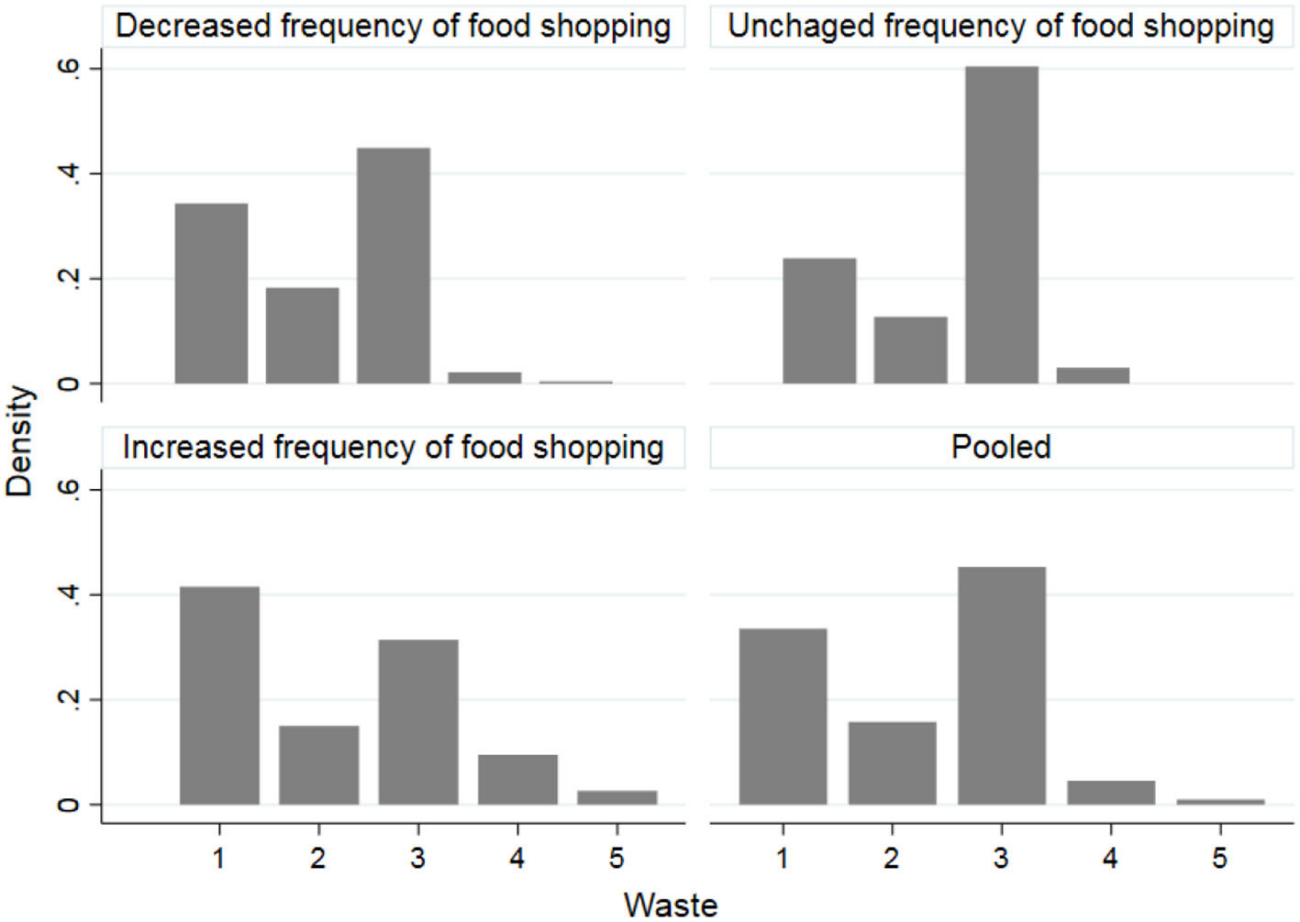
Relationships between food waste changes and frequency of food shopping during the lockdown for COVID-19 in Italy. 1 = food waste substantially decreased, 2 = food waste mildly decreased, 3 = food waste unchanged, 4 = food waste mildly increased, 5 = food waste substantially decreased.

The relationships between the variable *WASTE* and the variables indicating the amount of food purchased (*AM_DEC, AM_UNCHANGED, AM_INC*) and frequency of food shopping (*FREQ_DEC, FREQ_UNCHANGED, FREQ_INC*) are graphically presented in Figures 1, 2, respectively. The amount of food wasted has generally decreased even in cases where the amount of food purchased and the frequency of purchases has increased. There has also been an increase in food waste in cases where the amount of food purchased and to a greater extent the frequency of purchases has increased. In the latter case, the increase in food waste associated with the increase in the frequency of food purchased, could be due to the type of food purchased such as fresh products with a shorter shelf life. However, this result deserves further investigation.

Results from the estimation of the ordered logit model are reported in Table 3. A discussion of results is provided in terms of proportional odds ratios. For easier interpretation of the estimation results, odds ratios were obtained from the ordered logit coefficients. The odds ratios related to the variables *AM_DEC* (0.585, *p* < 0.01) and *FREQ_DEC* (0.718, *p* < 0.05) are statistically significant. This result suggests that respondents (i.e., main household food purchasers) who reduced the amount of purchased food (AM_DEC) and frequency of food shopping (FREQ_DEC) are less likely to substantially increase food waste during the Covid-19 emergency, with respect to those respondents who did not change the amount of purchased food and frequency of food shopping during the same period. It appears logical that consumers who reduced the amount of purchased food and the frequency of food shopping are less likely to increase their food waste.

**Table 3:** Ordered logit model results the interviewed sample^a^.

**Dep. var.: *WASTE***	**Coefficients**	**Odds ratios**	**Robust std. err**.
*AM_DEC*	−0.536^***^	0.585^***^	0.189
*AM_INC*	−0.424^***^	0.654^***^	0.136
*FREQ_DEC*	−0.331^**^	0.718^**^	0.135
*FREQ_INC*	−0.390^**^	0.677^**^	0.172
*female*	−0.083	0.921	0.109
*age*	−0.004	0.996	0.004
*south*	−0.350^**^	0.881^**^	0.152
*north*	−0.126	0.704	0.146
*island*	−0.080	0.923	0.210
*household*	−0.089^*^	0.915	0.054
*low_inc*	−0.545^*^	0.579^*^	0.328
*med_inc*	−0.254	0.775	0.366
*/cut1*	−2.382		0.466
*/cut2*	−1.700		0.464
*/cut3*	1.244		0.473
*/cut4*	3.065		0.553
Log Pseudo likelihood	−1,401.956		
Observation	1,188		

* *p < 0.10*,

** *p < 0.05*,

*** *p < 0.01*.

a *Robust standard error in brackets*.

*Data were collected during the lockdown for Covid-19 in Italy (9/3/2020-14/5/2020)*.

Similarly, the odds ratio associated with the variables *AM_HIGH* (0.654, *p* < 0.01) and *FREQ_HIGH* (0.677, *p* < 0.05) are statistically significant, indicating that respondents who increased the amount of purchased food and frequency of food shopping are less likely to report a substantial increase in their production of food waste during the Covid-19 emergency (with respect to those respondent who did not change the amount of purchased food and frequency of food shopping during the same period). While this finding may be counterintuitive as we would expect a positive relationship, it can be explained by changes in the food basket. Table 4 shows that respondents who increased the amount of purchased food and frequency of food shopping during the Covid-19 emergency increased their purchases of food with longer shelf-life such as pasta, rice, frozen and canned food, compared to consumers who decreased the amount of purchased food and frequency of food shopping during the pandemic.

**Table 4:** Summary statistics of scores for storage food during the lockdown for Covid-19 on a 5 points Likert scale.

**Variable**	**Obs**	**Mean**	**Std. dev**.	**Min**	**Max**	**Obs**	**Mean**	**Std. dev**.	**Min**	**Max**
***AM_DEC***						***AM_INC***				
*pasta*	164	2.756	1.022	1.000	5.000	728	3.736	0.772	1.000	5.000
*rice*	164	2.646	0.884	1.000	5.000	728	3.444	0.771	1.000	5.000
*frozen*	164	2.878	1.192	1.000	5.000	728	3.632	0.961	1.000	5.000
*canned*	164	2.909	1.061	1.000	5.000	728	3.549	0.959	1.000	5.000
***FREQ_DEC***						***FREQ_INC***				
**Variable**	**Obs**	**Mean**	**Std. dev**.	**Min**	**Max**	**Obs**	**Mean**	**Std. dev**.	**Min**	**Max**
*pasta*	510	3.316	0.940	1.000	5.000	347	3.899	0.752	1.000	5.000
*rice*	510	3.102	0.823	1.000	5.000	347	3.611	0.798	1.000	5.000
*frozen*	510	3.327	1.036	1.000	5.000	347	3.775	0.968	1.000	5.000
*canned*	510	3.324	0.967	1.000	5.000	347	3.608	1.024	1.000	5.000

*Data were collected during the lockdown for Covid-19 in Italy (9/3/2020-14/5/202)*.

Respondents' geographical location (i.e., region of the country) is also a significant factor in the likelihood of food waste increase during Covid-19. Reported changes in the production of food waste during the pandemic were equal among the Northern part of the country (*NORTH*), the Central part (*CENTER*) and the main islands (*ISLANDS*) (i.e., Sardinia and Sicily), while respondents from the Southern regions (*SOUTH*) were less likely to report an increase in their food waste production during the Covid-19 emergency with respect to the respondents from Central Italy (*CENTER*). This is indicated by the statistically significant odds ratio which is equal to 0.704 (*p* < 0.05).

These results may be driven by different forces. First, before the pandemic, residents in Central Italy reported producing more food waste than other regions. Annual urban waste production per-capita was 548, 517 and 449 Kg in Central, Northern and Southern Italy, respectively, in 2018 (24). Second, the Northern regions were affected the most by the pandemic (~190,000 total cases),^5^ followed by the Central regions (~30,000 total cases), while the least impacted areas were the Southern regions (~11,500 total cases) and the islands (~4,800 total cases) (25). These numbers suggest that the Southern regions were producing lower amounts of waste even before the pandemic and they were the least impacted by the Covid-19 emergency.^6^

The odds ratios of the variables *HOUSEHOLD* and *LOW_INC* are statistically significant (0.915 and 580, respectively) (*p* < 0.10), suggesting that an increase in the number of people in the household decreases the likelihood of reporting a substantial increase in food waste production during the Covid-19 emergency. Similarly, decreasing income also decreases the probability that food waste increases. In fact, our results indicate that low income respondents are less likely to report a substantial increase in food waste production during the lockdown than wealthier respondents (*MED_INC* and *HIGH_INC*). This result implies that tight budget constraints are associated with lower food waste. However, differences in food waste production that we found between low and high income households are not due to any of the motivational drivers investigated in the survey (Table 5). It may be due to other factors, for example, low income people have more awareness about food waste. This appears consistent with behavior exhibited during the economic crisis situation caused by the lockdown where the tendency of people, especially those on low incomes, is to reduce food waste and consume everything that has been purchased.^7^

**Table 5:** Motivations for food waste reduction between “low income” vs. “high income” reducers.

**Statement**	**Level of income^**a**^**	**Score**^****b****^					**Fisher test**
		**1**	**2**	**3**	**4**	**5**	
I want to ease the work of people in the waste collection	High income	8.3%	12.5%	16.7%	12.5%	50.0%	0.46
	Low income	12.0%	8.2%	26.2%	19.5%	34.1%	
I do not want to add pressure to the food management system	High income	8.3%	8.3%	29.2%	8.3%	45.8%	0.37
	Low income	7.5%	7.0%	24.0%	24.8%	36.8%	
I pay more attention because we live in a period of emergency.	High income	4.2%	4.2%	4.2%	8.3%	79.2%	0.25
	Low income	2.2%	1.0%	6.3%	18.0%	72.6%	
I buy less easily perishable food like salads or fruit.	High income	8.3%	0.0%	16.7%	33.3%	41.7%	0.48
	Low income	5.0%	7.9%	22.1%	25.0%	39.9%	

*Data were collected during the lockdown for Covid-19 in Italy (9/3/2020-14/5/2020)*.

a *Total 24 respondents with “high income” and 416 respondents with “low income”*.

b *Score 1 = I do not agree at all; Score 5 = I completely agree*.

For this paper, we also performed a qualitative analysis of additional motivational factors of changes in food waste production. Respondents who reported that they mildly or substantially decreased their food waste production during the lockdown were asked to indicate their agreement with a set of statements. Each statement reflects a reason for such decrease and respondents could add and rate additional motivating factors. An equivalent procedure was used for respondents who reported that they mildly or substantially increased their waste production during the lockdown, while respondents who reported an unchanged food waste production were not exposed to any follow-up question. The percentage of respondents who mildly (score = 4) and completely agreed (score = 5) to each statement is reported in Table 6.

**Table 6:** Motivation of decrease in food waste production during the lockdown for Covid-19.

**Statement**	**Percentage of agreement^**a**^**
I want to ease the work of waste collectors	52.99
I do not want to put pressure on the waste management system	61.36
I try to be careful because of COVID-19	90.94
I buy less perishable food	63.59

*Data were collected during the lockdown for Covid-19 in Italy (9/3/2020-14/5/2020)*.

a *Total 585 respondents who decreased food waste production. Percentages refer to respondents who mildly (score = 4) and completely agreed (score = 5) to each statement*.

The results suggest that the main factor driving the decrease in food waste production is a general concern regarding the impact that the pandemic could have on the waste management system (≈91%), followed by an increase in the purchasing of less perishable food (≈64%) e.g., pasta or canned food, and an intention not to add pressure to the whole waste management system (≈61%). Only 53% of the sample reported a specific intention to ease the work of waste collectors. Some respondents (12) suggested that they were sensitive to the problem even before the pandemic, while others stated that a reduction in food waste production is related to changes in their dietary and cooking habits as well as having more time to organize the cooking. Some stated financial constraints as a reason for a decrease in food waste production (Table 7).

**Table 7:** Additional reported motivating factors driving the decrease in food waste production.

**Additional statement**	**Number of respondents**
I used to recycle even before the pandemic	12
I changed my cooking style and use of leftovers	6
I have more time to dedicate to cooking	4
I change my diet during the pandemic	6
Financial constraints	4

*Data were collected during the lockdown for Covid-19 in Italy (9/3/2020-14/5/2020)*.

Only a small percentage of our sample (65 respondents, 5.5% of whole sample) indicated that their food waste increased during the pandemic (Table 8). About 74% of these respondents increased food waste production because of increased amount of food stock, followed by buying food which is easier to store (≈68%), and increasing the amount of food purchased (≈66%). About 62% reported that the increase in food waste production was caused by the increased amount of food they cooked. These results suggest that the increase in food waste, for the relatively small percentage of respondents in our sample, was likely due to stockpiling or panic buying phenomena that have driven consumers to buy more food than they needed. In fact, participants stated that they had increased food waste due to an increase in food purchases and food inventory at home, which therefore exceeded their real needs.

**Table 8:** Motivation of increase in food waste production during the lockdown for Covid-19.

**Statement**	**Percentage of agreement^**a**^**
The amount of food I buy has increased	66.15%
The amount of food I cook has increased	61.54%
The amount of food I stock has increased	73.85%
I buy food that is easier to store	67.69%

*Data were collected during the lockdown for Covid-19 in Italy (9/3/2020-14/5/2020)*.

a *Total 65 respondents who increased food waste production*.

## Discussion

The Covid-19 pandemic has played a significant role in changing the behavior of Italian consumers and in particular in their buying and eating habits. Our findings indicate that Italian consumers have increased the amount of food purchases during the Covid-19 lockdown when people's movements were severely restricted by the government to prevent the risk of contagion. This increase was influenced by the fear of disruptions of the food supply chain and the risk of being infected when going outside of home. We hypothesized that the increased amount of food purchases would increase the amount of food waste, but the results of our survey show that during the pandemic, the amount of food waste by Italian consumers actually decreased during Covid-19, at least during the period our survey was conducted. In other words, the increase in food purchases during the pandemic did not generally lead to an increase in food waste.

The decrease in food waste is related to the types of food purchased during the pandemic, which have been more directed toward non-perishable products such as canned and frozen food, pasta and rice. It is possible that consumers' risk aversion has caused not only a greater attention to food consumption habits but also a shift in consumers' purchasing habits that resulted in a decrease in household food waste. Interestingly, our results also suggest that the decrease in food waste was driven by the general concern about the impact the pandemic could have on the waste management system, and a tendency not to add pressure to the waste management system. If this result persists in the medium and long term, it could have important implications since reducing pressure on waste management systems could mean lowering food bills for households and disposal costs for restaurants, processors and farmers. However, future studies should examine this issue further and also in other geographical contexts.

An interesting result is related to the number of children in the household. Our study shows that in large families, food waste decreased during the lockdown, probably due to the fact that bigger households can better facilitate the re-use of leftovers (26). Moreover, this result could be driven by the fact that especially during the lockdown, the household food preparer has more time to organize and do the cooking as reported by a number of respondents in our survey.

Only a small percentage of our respondents indicated an increase in food waste due to increased food purchases and food stocks at home. This result is probably due to fears of supply chain disruptions that led to “panic buying” behaviors (20). It could also be due to a greater amount of fresh food purchases (e.g., fruit and vegetables) which increases the probability of producing food waste. This aspect should be explored further in future studies. However, our results suggest that the increase in food waste was a marginal phenomenon in Italy during the lockdown likely due to an increase in consumer awareness to make better use of available food supplies. It is then possible that reassurances from governments and the food industry about the resiliency of the food supply chain can further reduce the likelihood of panic buying and the amount of household food waste production.

Some limitations of our study should be highlighted such as the fact that our results are based on stated, not revealed behavior. Moreover, even if during the lockdown there were no significant interruptions in the food supply chain in Italy, our research cannot take into account changes in household food waste due to the closure of restaurants and other take-away food outlets. Furthermore, our results should be interpreted with some caution as food purchases with a longer shelf life do not necessarily imply a reduction in waste. In fact, non-perishable products are produced with a greater degree of processing and therefore it is possible that food waste is simply moved from one phase of the chain to another.

## Conclusions

Our study general shows that the amount of household food waste decreased in Italy during the Covid-19 lockdown despite an increase in the amount of food purchases. This result could suggest that the emergency period experienced in Italy during the lockdown increased awareness among the population to avoid or at least reduce food waste that has significant economic and environmental implications.

In conclusion, the Covid-19 pandemic is having significant impact on people's daily habits and economic activities. Interestingly, however, if there is a silver lining from an environmental perspective related to household food waste, it is that the pandemic has reduced the amount of food waste for a vast majority of households, at least in the Italian context. Our study is one of the first to examine food waste during Covid-19 pandemic, but more work is needed to fully understand food waste at the household level during emergency periods.

However, other aspects could be explored in future studies such as the trend of food waste during emergency periods among different socio-demographic groups i.e., young people vs. elderly, low income vs. high income, low education vs. high education, etc. Future research is also needed to assess whether the impact we found on food waste will persist in the longer term after Covid-19; i.e., whether it is actually transformational in nature. It would also be good to replicate our study in other countries to test the robustness of our findings.

## Data Availability Statement

The raw data supporting the conclusions of this article will be made available by the authors, without undue reservation.

## Ethics Statement

The studies involving human participants were reviewed and approved by Institutional Review Board (IRB) at the University of Arkansas – Fayetteville (USA). Written informed consent for participation was not required for this study in accordance with the national legislation and the institutional requirements.

## Author Contributions

SC and WY performed the computations. All authors conceived the presented idea, the analytical methods, discussed the results, and contributed to the final manuscript.

## Conflict of Interest

The authors declare that the research was conducted in the absence of any commercial or financial relationships that could be construed as a potential conflict of interest.

## Footnotes

^1^The management of food waste in Italy began in 1972 with Presidential Decree of 26 October 1972, n. 633 - Establishment and implementation of a value added tax and has moved progressively up the Connected to the Environmental Stability Law of 2014 (“Provisions on environmental measures to promote a green economy and to contain the excessive use of natural resources”) and to the national strategy to prevent and reduce food waste. This current legislation in Italy includes separate food waste bins, frequency of collection and payment methods for waste collection by households. Moreover, the development of voluntary agreements at local level (e.g., provinces or municipalities) to involve stakeholders along the food supply chain was an additional step to reduce and prevent food waste in Italy.

^2^The sample was generally stratified using gender, age, income, education.

^3^Likert scales used in our research for food waste statements are not validated scales.

^4^Other variables were not included because of multi-collinearity issues.

^5^Total cases are the number of deaths, positives and recovered.

^6^The resiliency of waste management systems is very heterogeneous across regions. For example, the rate of recycling of urban waste in the South is approximately 20 percentage points lower than in the North. However, this discussion is beyond the scope of this paper since we focus on food waste production rather than food waste management in this paper.

^7^A Brant test failed to reject the proportional odds assumption assumed by ordered logit models (χ^2^ = −3175.76)
