# Supplementary material for: Impact of Covid-19 on Household Food Waste: The Case of Italy
Source: Front Nutr. 2020 Dec 2;7:585090. doi: 10.3389/fnut.2020.585090 (PMC7738320; doi:10.3389/fnut.2020.585090)
Supplement: Supplementary file 1 [file Table_1.DOCX]

**Survey title: Food consumption behavior during COVID-19 pandemic**

**INVITATION TO PARTICIPATE**

You are invited to participate in a research study about food purchases related to the coronavirus. Please read through the following information and indicate your consent below to begin the survey.

**INFORMATION ABOUT THE RESEARCH STUDY**

The survey should take approximately 15 minutes.

**You must be 18 years of age or older to participate**

 Risks and Benefits:  Your participation will assist in advancement of knowledge of Italy consumer's choice behavior. There are no anticipated risks to participating in this study.

 Voluntary Participation:  Your participation in the research is completely voluntary.

 Confidentiality:  Your responses on the survey will be recorded anonymously. No identifying personal information will be collected in the survey. Only basic demographic information (age, gender, education etc.) will be collected.

 Right to Withdraw:  You are free to refuse to participate in the research and to stop filling out the survey at any time. If you have questions or concerns about this study, you may contact xxxx. For questions or concerns about your rights as a research participant, please contact xxxx.

**Thank you for your participation!**

By clicking the button below and taking the survey, you acknowledge that you have read the above statement and have been able to ask questions and express concerns, which have been satisfactorily answered by the investigator. You understand the purpose of the study as well as the potential risks and benefits that are involved. You understand that your participation is voluntary and that no rights have been waived in giving your consent.  You acknowledge that you are 18 years of age or older and that you may choose to terminate your participation in the study at any time for any reason.

I consent, begin the study I do not consent, I do not wish to participate

We are conducting a survey to learn about food consumption behavior during COVID-19 pandemic in Italy.

The goal of the survey is to understand how a pandemic like the novel coronavirus affects food consumption behavior. The findings can help policy makers, communities and companies in emergency management efforts.

Your responses are completely anonymous and cannot be linked to you in any way, shape, or form. The information collected here will not be used for any purpose other than this study.

A. Are you of legal age (over18)?

YES

NO

B. Are you the main food purchaser in your household?

YES

NO

C. Do you commit to carefully reading and providing thoughtful and accurate answers to the questions in this survey?

I will read carefully and provide my best answers

I will not read carefully and provide my best answers

I cannot promise either way

The survey will consist of three parts. In part 1, you will be asked a set of questions regarding your food purchasing behavior before and during the coronavirus pandemic. In part 2, you will be asked few questions regarding the importance that you place on several food attributes before and after the coronavirus pandemic. In part 3, you will be asked few questions regarding your socio-demographic status.

**PART 1**

**Questions on visits to the supermarket**

1. Since the coronavirus pandemic that began in February, has the frequency with which you buy food increased, decreased or remained unchanged? Please rate on a scale from 1 (decreased substantially) to 5 (increased substantially)?

| 1  (decreased substantially) | 2  (decreased mildly) | 3  (unchanged) | 4  (increased  mildly) | 5  (increased  substantially) |
| --- | --- | --- | --- | --- |
| □ | □ | □ | □ | □ |

2. BEFORE the coronavirus pandemic that began in February, how often did you do food shopping? (choose only one answer)

Every day

Once every 2 days

Twice a week

Once a week

Every 2 weeks

Once a month

3. SINCE the coronavirus pandemic that began in February, how often do you do food shopping? (choose only one answer)

Every day

Once every 2 days

Twice a week

Once a week

Every 2 weeks

Once a month

3a. If you increased the frequency of your food purchases during the coronavirus pandemic (with respect to before the pandemic), please rate your level of agreement, on a scale from 1 (I do not agree at all) to 5 (I completely agree), with the statements below:

| I increased the frequency of my food purchases because: | 1  I do not agree at all | 2 | 3 | 4 | 5  I completely agree |
| --- | --- | --- | --- | --- | --- |
| The amount of food I eat has increased since the pandemic began | □ | □ | □ | □ | □ |
| The likelihood of catching the coronavirus in the supermarket is low | □ | □ | □ | □ | □ |
| I am afraid there will be disruptions in the food supply | □ | □ | □ | □ | □ |
| I cook more and order less take-away food than we used to | □ | □ | □ | □ | □ |
| I tend to stock more food than we used to | □ | □ | □ | □ | □ |
| Others: please specify  _---------------- |  |  |  |  |  |

3b. If you decreased the frequency of your food purchases during the coronavirus pandemic (with respect to before the pandemic), please rate your level of agreement, on a scale from 1 (I do not agree at all) to 5 (I completely agree), with the statements below:

| I decreased the frequency of our food purchases because: | 1  I do not agree at all | 2 | 3 | 4 | 5  I completely agree |
| --- | --- | --- | --- | --- | --- |
| Fear of catching the coronavirus in the supermarket | □ | □ | □ | □ | □ |
| Respect the guidelines on lockdown provided by the Government | □ | □ | □ | □ | □ |
| Protect other peoples’ health and the health care system | □ | □ | □ | □ | □ |
| Standing in the cue outside the supermarket is boring | □ | □ | □ | □ | □ |
| I am struggling financially | □ | □ | □ | □ | □ |
| Others: please specify  _---------------- |  |  |  |  |  |

**Questions on purchasing behavior**

4. Since the coronavirus pandemic that began in February, has the amount of food you buy each time you leave home and go to the market increased, decreased or remained unchanged? Please rate on a scale from 1 (decreased substantially) to 5 (increased substantially)?

| 1  (decreased substantially) | 2  (decreased mildly) | 3  (unchanged) | 4  (increased  mildly) | 5  (increased  substantially) |
| --- | --- | --- | --- | --- |
| □ | □ | □ | □ | □ |

5. Since the coronavirus pandemic that began in February, has your purchase of the following foods increased, decreased, or remained unchanged? Please rate on a scale from 1 (decreased substantially) to 5 (increased substantially)?

| **Types of food** | 1  (decreased substantially) | 2  (decreased mildly) | 3  (unchanged) | 4  (increased  mildly) | 5  (increased  substantially) |
| --- | --- | --- | --- | --- | --- |
| Pasta | □ | □ | □ | □ | □ |
| Rice | □ | □ | □ | □ | □ |
| Flour | □ | □ | □ | □ | □ |
| Olive oil & Vinegar | □ | □ | □ | □ | □ |
| Bread and bakery products | □ | □ | □ | □ | □ |
| Meat, fish, eggs | □ | □ | □ | □ | □ |
| Milk and dairy products (i.e. butter) | □ | □ | □ | □ | □ |
| Frozen foods | □ | □ | □ | □ | □ |
| Canned foods | □ | □ | □ | □ | □ |
| Ready meals | □ | □ | □ | □ | □ |
| Vegetables, fruit | □ | □ | □ | □ | □ |
| Snacks, cookies | □ | □ | □ | □ | □ |
| Organic products | □ | □ | □ | □ | □ |
| Gluten- and Lactose-free products | □ | □ | □ | □ | □ |
| Bottled water | □ | □ | □ | □ | □ |
| Soft drinks | □ | □ | □ | □ | □ |
| Alcoholic beverages | □ | □ | □ | □ | □ |
| Others: please specify |  |  |  |  |  |

**Questions on food expenditures**

6. Since the coronavirus pandemic began in February, has the amount of money you spend on food (not including eating out) increased, decreased or remained unchanged? Please rate on a scale from 1 (decreased substantially) to 5 (increased substantially)?

| 1  (decreased substantially) | 2  (decreased mildly) | 3  (unchanged) | 4  (increased  mildly) | 5  (increased  substantially) |
| --- | --- | --- | --- | --- |
| □ | □ | □ | □ | □ |

6a. If the amount of money you spend on food (not including eating out) during the coronavirus pandemic has increased, please rate your level of agreement, on a scale from 1 (I do not agree at all) to 5 (I completely agree), with the statements below:

| The amount of money I spend on food has increased because: | 1  I do not agree at all | 2 | 3 | 4 | 5  I completely agree |
| --- | --- | --- | --- | --- | --- |
| I tend to buy more food | □ | □ | □ | □ | □ |
| I cook less and order more take-away food than we used to | □ | □ | □ | □ | □ |
| I tend to do more online food shopping | □ | □ | □ | □ | □ |
| I tend to buy more ready-meals | □ | □ | □ | □ | □ |
| Prices have increased | □ | □ | □ | □ | □ |
| I tend to stock more food than we used to | □ | □ | □ | □ | □ |
| Others: please specify  _---------------- |  |  |  |  |  |

6b. If the amount of money you spend on food (not including eating out) during the coronavirus pandemic has decreased, please rate your level of agreement, on a scale from 1 (I do not agree at all) to 5 (I completely agree), with the statements below:

| The amount of money I spend on food has decreased because: | 1  I do not agree at all | 2 | 3 | 4 | 5  I completely agree |
| --- | --- | --- | --- | --- | --- |
| I tend to buy less food | □ | □ | □ | □ | □ |
| I cook more and order less take-away food than we used to | □ | □ | □ | □ | □ |
| I tend to do more online food shopping | □ | □ | □ | □ | □ |
| I tend to buy less ready-meals | □ | □ | □ | □ | □ |
| I cook more from scratches and buy less prepared food | □ | □ | □ | □ | □ |
| Prices have decreased | □ | □ | □ | □ | □ |
| I tend to stock more food than we used to | □ | □ | □ | □ | □ |
| I am struggling financially |  |  |  |  |  |
| Others: please specify  _---------------- |  |  |  |  |  |

7. Since the coronavirus epidemic began in February, has your expenditure for the following foods increased, decreased, or remained unchanged? Please rate on a scale from 1 (decreased substantially) to 5 (increased substantially)?

| **Types of food** | 1  (decreased substantially) | 2  (decreased mildly) | 3  (unchanged) | 4  (increased  mildly) | 5  (increased  substantially) |
| --- | --- | --- | --- | --- | --- |
| Pasta | □ | □ | □ | □ | □ |
| Rice | □ | □ | □ | □ | □ |
| Flour | □ | □ | □ | □ | □ |
| Olive oil & Vinegar | □ | □ | □ | □ | □ |
| Bread and bakery products | □ | □ | □ | □ | □ |
| Meat, fish, eggs | □ | □ | □ | □ | □ |
| Milk and dairy products (i.e. butter) | □ | □ | □ | □ | □ |
| Frozen foods | □ | □ | □ | □ | □ |
| Canned foods | □ | □ | □ | □ | □ |
| Ready meals | □ | □ | □ | □ | □ |
| Vegetables, fruit | □ | □ | □ | □ | □ |
| Snacks, cookies | □ | □ | □ | □ | □ |
| Organic products | □ | □ | □ | □ | □ |
| Gluten- and Lactose-free products | □ | □ | □ | □ | □ |
| Bottled water | □ | □ | □ | □ | □ |
| Soft drinks | □ | □ | □ | □ | □ |
| Alcoholic beverages | □ | □ | □ | □ | □ |
| Others: please specify |  |  |  |  |  |

**Questions on food stocking**

8. Since the coronavirus pandemic began in February, has the amount of food you stock in your house increased, decreased or remained unchanged? Please rate on a scale from 1 (decreased substantially) to 5 (increased substantially)?

| 1  (decreased substantially) | 2  (decreased mildly) | 3  (unchanged) | 4  (increased  mildly) | 5  (increased  substantially) |
| --- | --- | --- | --- | --- |
| □ | □ | □ | □ | □ |

8a. If the amount of food you stock (not including eating out) during the coronavirus pandemic has increased, please rate your level of agreement, on a scale from 1 (I do not agree at all) to 5 (I completely agree), with the statements below:

| The amount of food I stock has increased because: | 1  I do not agree at all | 2 | 3 | 4 | 5  I completely agree |
| --- | --- | --- | --- | --- | --- |
| I tend to buy more food | □ | □ | □ | □ | □ |
| I tend to eat more | □ | □ | □ | □ | □ |
| I tend to do more online food shopping | □ | □ | □ | □ | □ |
| I tend to be more concerned about food supply issues | □ | □ | □ | □ | □ |
| Others: please specify  _---------------- |  |  |  |  |  |

8b. If the amount of food you stock (not including eating out) during the coronavirus pandemic has decreased, please rate your level of agreement, on a scale from 1 (I do not agree at all) to 5 (I completely agree), with the statements below:

| The amount of food I stock has decreased because: | 1  I do not agree at all | 2 | 3 | 4 | 5  I completely agree |
| --- | --- | --- | --- | --- | --- |
| I tend to buy less food | □ | □ | □ | □ | □ |
| I tend to eat less | □ | □ | □ | □ | □ |
| I tend to buy what is necessary | □ | □ | □ | □ | □ |
| I am struggling financially | □ | □ | □ | □ | □ |
| Others: please specify  _---------------- |  |  |  |  |  |

**Question on groceries and eating habits**

9. Since the coronavirus epidemic began in February, has your purchase in the following food retailers increased, decreased or remained unchanged? Please rate on a scale from 1 (decreased substantially) to 5 (increased substantially)?

| **Food retailers** | 1  (decreased substantially) | 2  (decreased mildly) | 3  (unchanged) | 4  (increased  mildly) | 5  (increased  substantially) |
| --- | --- | --- | --- | --- | --- |
| Hypermarket/supermarket | □ | □ | □ | □ | □ |
| Mini market/small market (butcheries, dairies, bakeries, etc.) | □ | □ | □ | □ | □ |
| At the market (once a week/daily) | □ | □ | □ | □ | □ |
| Online with home delivery | □ | □ | □ | □ | □ |

10. BEFORE the coronavirus epidemic began in February, how often did you buy food online?

Every day

Once every 2 days

Twice a week

Once a week

Every 2 weeks

Once a month

Never

11. SINCE the coronavirus epidemic began in February, how often did you buy food online?

Every day

Once every 2 days

Twice a week

Once a week

Every 2 weeks

Once a month

Never

12. Have you experienced shortage of some food products in the retail stores during the coronavirus epidemic?

Yes

No

13. What kind of food did you experience a shortage of during the coronavirus epidemic?

| **Types of food** | **YES** | **NO** |
| --- | --- | --- |
| Pasta | □ | □ |
| Rice | □ | □ |
| Flour | □ | □ |
| Olive oil & Vinegar | □ | □ |
| Bread and bakery products | □ | □ |
| Meat, fish, eggs | □ | □ |
| Milk and dairy products (i.e. butter) | □ | □ |
| Frozen foods | □ | □ |
| Canned foods | □ | □ |
| Ready meals | □ | □ |
| Vegetables, fruit | □ | □ |
| Organic products | □ | □ |
| Gluten- and Lactose-free products | □ | □ |
| Bottled water | □ | □ |
| Soft drinks | □ | □ |
| Alchoolic beverages | □ | □ |
| Snacks, cookies | □ | □ |
| Others: please specify |  |  |

14. Have you acquired food from food banks or food pantries during the coronavirus crisis?

Yes

No

**Questions of food waste**

15. Since the coronavirus epidemic began in February, has the amount of your food waste (i.e., food that is not consumed and is wasted) increased, decreased or remained unchanged? Please rate on a scale from 1 (decreased substantially) to 5 (increased substantially)?

| 1  (decreased substantially) | 2  (decreased mildly) | 3  (unchanged) | 4  (increased  mildly) | 5  (increased  substantially) |
| --- | --- | --- | --- | --- |
| □ | □ | □ | □ | □ |

15a. If your food waste has increased during the coronavirus pandemic (with respect to before the pandemic), please rate your level of agreement, on a scale from 1 (I do not agree at all) to 5 (I completely agree), with the statements below:

| The amount of my food waste has increased because: | 1  I do not agree at all | 2 | 3 | 4 | 5  I completely agree |
| --- | --- | --- | --- | --- | --- |
| The amount of food I buy has increased | □ | □ | □ | □ | □ |
| The amount of food I cook has increased | □ | □ | □ | □ | □ |
| The amount of food I stock has increased | □ | □ | □ | □ | □ |
| I buy food that is easier to store, such as canned food. | □ | □ | □ | □ | □ |
| Others: please specify  _---------------- |  |  |  |  |  |

15b. If your food waste has decreased during the coronavirus pandemic (with respect to before the pandemic), please rate your level of agreement, on a scale from 1 (I do not agree at all) to 5 (I completely agree), with the statements below:

| The amount of my food waste has decreased because: | 1  I do not agree at all | 2 | 3 | 4 | 5  I completely agree |
| --- | --- | --- | --- | --- | --- |
| I want to ease the work of people in the waste collection | □ | □ | □ | □ | □ |
| I do not want to add pressure to the food management system | □ | □ | □ | □ | □ |
| I pay more attention because we live in a period of emergency. | □ | □ | □ | □ | □ |
| I buy less easily perishable food like salads or fruit. | □ | □ | □ | □ | □ |
| Others: please specify  ---------------- |  |  |  |  |  |

**Questions on lifestyles**

16. Since the coronavirus epidemic began in February, has the amount of time you spend on the following activities increased, decreased or remained unchanged? Please rate on a scale from 1 (decreased substantially) to 5 (increased substantially)?

| Activity | 1  (decreased substantially) | 2  (decreased mildly) | 3  (unchanged) | 4  (increased  mildly) | 5  (increased  substantially) |
| --- | --- | --- | --- | --- | --- |
| Hours of sleep | □ | □ | □ | □ | □ |
| Physical Exercising | □ | □ | □ | □ | □ |
| Time spent at work | □ | □ | □ | □ | □ |
| Reading | □ | □ | □ | □ | □ |
| Eating | □ | □ | □ | □ | □ |
| Drinking alcohol | □ | □ | □ | □ | □ |
| Watching TV | □ | □ | □ | □ | □ |
| Playing videogames | □ | □ | □ | □ | □ |
| Social media (facebook, twitter, Instagram etc.) | □ | □ | □ | □ | □ |
| Calls and texting | □ | □ | □ | □ | □ |
| Gardening | □ | □ | □ | □ | □ |
| House work | □ | □ | □ | □ | □ |
| Cleaning the house | □ | □ | □ | □ | □ |

**PART 2**

[In the part, we have three treatment groups. Information given in each group was shown below]

**TREATMENT GROUPS**

**1 NO-INFO GROUP:**

In this section, you will be asked twelve choice questions that will provide information regarding the relative importance that you place on different food attributes.

For each question below, we ask you to indicate which attribute you consider to be the most important and which attribute you consider to be the least important when you buy foods. Please, check for each question just one attribute that you consider as the most important and just one attribute that you consider as the least important.

Please carefully and truthfully answer each of the questions below since your responses are important to us and can help policy makers, communities, and companies in emergency management efforts. Thank you.

**2 NEGATIVE INFO GROUP:**

In this section, you will be asked twelve choice questions that will provide information regarding the relative importance that you place on different food attributes.

For each question below, we ask you to indicate which attribute you consider to be the most important and which attribute you consider to be the least important when you buy foods. Please, check for each question just one attribute that you consider as the most important and just one attribute that you consider as the least important.

**Before asking you to make choices, we provide you with some information regarding the likelihood of food supply chain disruptions due to the coronavirus pandemic**.

Food Supply chains are a complex web of interactions and international actors: producers, processors, distributors, retailers etc.. As the coronavirus spreads and lockdowns increase, there are many ways the food supply chains could be tested and strained in the coming weeks and months: i) production of food could decrease because of labor shortages due to deaths, sickness, and quarantines and ii) imports and exports could decrease because of logistical issues in food transportation due to coronavirus related restrictions. **Today, there are no supply shocks in terms of food availability, but experts forecast that disruptions in the food supply chain can occur in the next two months.**

Please carefully and truthfully answer each of the questions below since your responses are important to us and can help policy makers, communities, and companies in emergency management efforts. Thank you.

**3 AMBIGUITY INFO GROUP:**

In this section, you will be asked twelve choice questions that will provide information regarding the relative importance that you place on different food attributes.

For each question below, we ask you to indicate which attribute you consider to be the most important and which attribute you consider to be the least important when you buy foods. Please, check for each question just one attribute that you consider as the most important and just one attribute that you consider as the least important.

**Before asking you to make choices, we provide you with some information regarding the likelihood of food supply chain disruptions due to the coronavirus pandemic**.

Food Supply chains are a complex web of interactions and international actors: producers, processors, distributors, retailers etc.. As the coronavirus spreads and lockdowns increase, there are many ways the food supply chains could be tested and strained in the coming weeks and months: i) production of food could decrease because of labor shortages due to deaths, sickness, and quarantines and ii) imports and exports could decrease because of logistical issues in food transportation due to coronavirus related restrictions. **Today, there are no supply shock in terms of food availability. Some experts forecast that disruptions in the food supply chain can occur in the next two months but other experts do not forecast such disruptions in the food supply chain.**

Please carefully and truthfully answer each of the questions below since your responses are important to us and can help policy makers, communities, and companies in emergency management efforts. Thank you.

**17. Which of the following attributes is most important and which is least important when you purchase food? Please, check only one attribute as the most important and only one attribute as the least important**

| Most Important  ONE ANSWER | Attribute | Least Important  ONE ANSWER |
| --- | --- | --- |
|  | Appearance  (the food looks appealing and appetizing) |  |
|  | Novelty  (the food is something new that you have not tried before) |  |
|  | Fairness  (farmers, processors and retailers get a fair share of the price) |  |
|  | Origin  (whether the food is produced locally, in Italy, or abroad) |  |

**18. Which of the following attributes is most important and which is least important when you purchase food? Please, check only one attribute as the most important and only one attribute as the least important**

| Most Important  ONE ANSWER | Attribute | Least Important  ONE ANSWER |
| --- | --- | --- |
|  | Appearance  (the food looks appealing and appetizing) |  |
|  | Convenience  (how easy and fast the food is to cook and eat) |  |
|  | Taste  (the flavor of the food in your mouth) |  |
|  | Animal welfare  (well-being of farm animals) |  |

**19. Which of the following attributes is most important and which is least important when you purchase food? Please, check only one attribute as the most important and only one attribute as the least important**

| Most Important  ONE ANSWER | Attribute | Least Important  ONE ANSWER |
| --- | --- | --- |
|  | Naturalness  (made without modern food technologies like genetic engineering, hormone treatment and food irradiation) |  |
|  | Nutrition  (amount and type of fat, protein, etc.) |  |
|  | Appearance  (the food looks appealing and appetizing) |  |
|  | Price  (price you pay for the food) |  |

**20. Which of the following attributes is most important and which is least important when you purchase food? Please, check only one attribute as the most important and only one attribute as the least important**

| Most Important  ONE ANSWER | Attribute | Least Important  ONE ANSWER |
| --- | --- | --- |
|  | Safety  (eating the food will not make you sick) |  |
|  | Novelty  (the food is something new that you have not tried before) |  |
|  | Convenience  (how easy and fast the food is to cook and eat) |  |
|  | Nutrition  (amount and type of fat, protein, etc.) |  |

**21. Which of the following attributes is most important and which is least important when you purchase food? Please, check only one attribute as the most important and only one attribute as the least important**

| Most Important  ONE ANSWER | Attribute | Least Important  ONE ANSWER |
| --- | --- | --- |
|  | Taste  (the flavor of the food in your mouth) |  |
|  | Novelty  (the food is something new that you have not tried before) |  |
|  | Animal welfare  (well-being of farm animals) |  |
|  | Naturalness  (made without modern food technologies like genetic engineering, hormone treatment and food irradiation) |  |

**22. Which of the following attributes is most important and which is least important when you purchase food? Please, check only one attribute as the most important and only one attribute as the least important**

| Most Important  ONE ANSWER | Attribute | Least Important  ONE ANSWER |
| --- | --- | --- |
|  | Environmental impact  (effects of food production on the environment) |  |
|  | Naturalness  (made without modern food technologies like genetic engineering, hormone treatment and food irradiation) |  |
|  | Safety  (eating the food will not make you sick) |  |
|  | Appearance  (the food looks appealing and appetizing) |  |

**23. Which of the following attributes is most important and which is least important when you purchase food? Please, check only one attribute as the most important and only one attribute as the least important**

| Most Important  ONE ANSWER | Attribute | Least Important  ONE ANSWER |
| --- | --- | --- |
|  | Origin  (whether the food is produced locally, in Italy or abroad) |  |
|  | Price  (price you pay for the food) |  |
|  | Safety  (eating the food will not make you sick) |  |
|  | Taste  (the flavor of the food in your mouth) |  |

**24 Which of the following attributes is most important and which is least important when you purchase food? Please, check only one attribute as the most important and only one attribute as the least important**

| Most Important  ONE ANSWER | Attribute | Least Important  ONE ANSWER |
| --- | --- | --- |
|  | Novelty  (the food is something new that you have not tried before) |  |
|  | Price  (price you pay for the food) |  |
|  | Convenience  (how easy and fast the food is to cook and eat) |  |
|  | Environmental impact  (effects of food production on the environment) |  |

**25. Which of the following attributes is most important and which is least important when you purchase food? Please, check only one attribute as the most important and only one attribute as the least important**

| Most Important  ONE ANSWER | Attribute | Least Important  ONE ANSWER |
| --- | --- | --- |
|  | Origin  (whether the food is produced locally, in Italy or abroad) |  |
|  | Environmental impact  (effects of food production on the environment) |  |
|  | Animal welfare  (well-being of farm animals) |  |
|  | Nutrition  (amount and type of fat, protein, etc.) |  |

**26. Which of the following attributes is most important and which is least important when you purchase food? Please, check only one attribute as the most important and only one attribute as the least important**

| Most Important  ONE ANSWER | Attribute | Least Important  ONE ANSWER |
| --- | --- | --- |
|  | Fairness  (farmers, processors and retailers get a fair share of the price) |  |
|  | Naturalness  (made without modern food technologies like genetic engineering, hormone treatment and food irradiation) |  |
|  | Origin  (whether the food is produced locally, in Italy or abroad) |  |
|  | Convenience  (how easy and fast the food is to cook and eat) |  |

**27. Which of the following attributes is most important and which is least important when you purchase food? Please, check only one attribute as the most important and only one attribute as the least important**

| Most Important  ONE ANSWER | Attribute | Least Important  ONE ANSWER |
| --- | --- | --- |
|  | Animal welfare  (well-being of farm animals) |  |
|  | Safety  (eating the food will not make you sick) |  |
|  | Fairness  (farmers, processors and retailers get a fair share of the price) |  |
|  | Price  (price you pay for the food) |  |

**28. Which of the following attributes is most important and which is least important when you purchase food? Please, check only one attribute as the most important and only one attribute as the least important**

| Most Important  ONE ANSWER | Attribute | Least Important  ONE ANSWER |
| --- | --- | --- |
|  | Environmental impact  (effects of food production on the environment) |  |
|  | Taste  (the flavor of the food in your mouth) |  |
|  | Fairness  (farmers, processors and retailers get a fair share of the price) |  |
|  | Nutrition  (amount and type of fat, protein, etc.) |  |

**PART 3**

Finally, we would like some background information about you. This is an important part of our analysis. The survey is anonymous, and your name is in no way linked to the responses.

**29. Gender:**

Male

Female

|  | NUMBER OF YEARS |
| --- | --- |
| **30: What is your age?** |  |

| **31: Which part of Italy do you live in?** | ONE ANSWER |
| --- | --- |
| North |  |
| Center |  |
| South |  |
| Islands |  |

| **32: Do you live in an urban or rural area?** | ONE ANSWER |
| --- | --- |
| In a city with more than 100.000 inhabitants |  |
| In a city or urban area with between 1,000 and 99,999 inhabitants |  |
| In a smaller town with less than 1000 inhabitants |  |

| **33: Are you ...** | ONE ANSWER |
| --- | --- |
| Married |  |
| Cohabitant |  |
| Unmarried/ Never been married |  |
| Previously married/ Separated/ Divorced |  |
| Widow/ Widower |  |

| **34: How many children who are 18 years old or younger live in your household?** | ONE ANSWER |
| --- | --- |
| No children |  |
| One child |  |
| Two children |  |
| Three children |  |
| Four children |  |
| More than four children |  |

|  | Number of persons |
| --- | --- |
| **35: How many persons with paid work live in your household?** |  |

|  | Yes | No |
| --- | --- | --- |
| **36: Do you live, or have you ever lived on a farm?** |  |  |

| **37: Was one or both of your parents born outside Italy?** | ONE ANSWER |
| --- | --- |
| Both were born in Italy |  |
| One of them was born outside Italy |  |
| Both of them were born outside Italy |  |
| I do not know |  |

**38. What is your highest level of education?**

Elementary school

Middle school

High school

Degree

Post-degree (Phd, Master)

**39. Which of the following best describes your yearly household’s net income:**

Less than €20.000

€20.000 to €39.999

€40.000 to €59.999

€60.000 to €79.999

€80.000 to €99.999

At least €100.000

**40. How many members does your household have?** _________________________

**41. How many individuals age 65 and over live in your household?** _________________________

**42. Do you have any food allergies?**

Yes If yes, which ones?

No

**43. Have you lost your job because of the coronavirus epidemic?**

Yes

No

**44. Are you in cassa integrazione?**

Yes

No

**45. Are you receiving other form of benefits (for example food stamps)?**

Yes

No

**46. In your opinion, the incidence or number of coronavirus infections in the city where you are residing during the coronavirus pandemic is:**

Very High

High

Medium

Low

Very Low
